# The activity of the ribonucleotide monophosphatase UmpH is controlled by interaction with the GlnK signaling protein in *Escherichia coli*

**DOI:** 10.1016/j.jbc.2024.107931

**Published:** 2024-10-24

**Authors:** Ana Carolina Aparecida Gonçalves, Tatiana de Mello Damasco Nunes, Erick Parize, Edileusa Cristina Marques Gerhardt, Gustavo Antônio de Souza, Jörg Scholl, Karl Forchhammer, Luciano Fernandes Huergo

**Affiliations:** 1 Setor Litoral, UFPR Matinhos, Paraná, Brazil; 2 Programa de Pós-Graduação em Ciências - Bioquímica, UFPR Curitiba, Paraná, Brazil; 3 Department of Biochemistry, Universidade Federal do Rio Grande do Norte, Natal, Rio Grande do Norte, Brazil; 4 Interfakultäres Institut für Mikrobiologie und Infektionsmedizin der Eberhard-Karls Universität Tübingen, Tübingen, Germany

**Keywords:** PII protein, UmpH, metabolic regulation, protein–protein interaction, nutrient sensing, allosteric regulation, 2-oxoglutarate, bacterial metabolism

## Abstract

The PII signaling proteins are ubiquitous in prokaryotes serving as crucial metabolic hubs in different metabolic pathways because of their ability to sense and integrate signals of the cellular nitrogen, carbon, and energy levels. In this study, we used ligand fishing assays to identify the ribonucleotide monophosphatase UmpH enzyme as a novel target of the PII signaling protein GlnK in *Escherichia coli*. *In vitro* analyses showed that UmpH interacts specifically with the PII protein GlnK but not with its paralog protein GlnB. The UmpH–GlnK complex is modulated by the GlnK uridylylation status and by the levels of the GlnK allosteric effectors ATP, ADP, and 2-oxoglutarate. Upon engaging interaction with GlnK, UmpH becomes less active toward its substrate uridine 5′-monophosphate. We suggest a model where GlnK will physically interact to reduce the UmpH activity during the transition from N-starvation to N-sufficient conditions. Such a mechanism may help the cells to reprogram the fate of uridine 5′-monophosphate from catabolism to anabolism avoiding futile cycling of key nutrients.

The regulation of metabolism is vital for the fitness of microbes. Free-living bacteria are constantly challenged with changes in the availability of nutrients in the environment and must, therefore, rapidly adjust the flow on different metabolic pathways (1). PII proteins play a central role in metabolic regulation and are ubiquitous in nature being present in bacteria, archaea, and plants (2). The PII proteins act as a metabolic hub, sensing the nutritional status of the cell and transducing this information to regulate a range of metabolic pathways (2, 3).

The proteins of the PII family were discovered in 1968 by Bennett Shapiro, while carrying out experiments to discover how the enzyme glutamine synthetase, a key player in bacterial nitrogen metabolism, was regulated (4). Since its initial discovery, it became clear that PII homologs are widespread in nature being found in bacteria, archaea, and in eukaryotic phototrophs (3). Canonical PII proteins (which are similar to the original PII described by Shapiro) are divided into four subgroups, *glnB*, *glnK*, *nifI*, and PII-New, according to the conservation of genetic linkage and similarity at the amino acid sequence level (5, 6). In most cases, GlnB has its encoding gene linked to *glnA* (the glutamine synthetase encoding gene) or *nadE* (encoding NAD synthetase) and is mostly found in proteobacteria and cyanobacteria; GlnK is encoded by a gene linked to *amtB* (encoding an ammonia channel); NifI is encoded by a gene that is linked to the *nif* (encoding nitrogenase subunits); PII-New group genes are present in proteobacteria and some bacteroidetes and are found linked to genes related to heavy metal efflux pumps (5, 6).

Canonical PII proteins are homotrimers that are highly conserved in sequence and structure (7, 8). The structure of a canonical PII protein consists of a core barrel-like structure from where three loops emerge from each subunit, namely the T, C, and B loops (2). The T-loop is the most prominent; it is well exposed to the solvent and may be subject to post-translational covalent modifications (8, 9, 10). In proteobacteria, the Y51 residue of the T-loop is subject to reversible uridylation (9, 10, 11, 12).

The genome of *Escherichia coli* encodes two PII proteins, GlnB (product of the *glnB* gene) and GlnK (product of the *glnK* gene). The GlnB and GlnK proteins are 67% identical in sequence and are structurally similar (13, 14). Given the high similarity, GlnB and GlnK paralogs have some overlapping but also specific functions (15, 16, 17). While the *glnB* gene is constitutively expressed, the *glnK* gene is induced upon nitrogen starvation, which indicates that the GlnK function is required during nitrogen starvation (14). PII proteins are subjected in a similar way to reversible covalent modification through a bifunctional (uridylyl-transferase/removing) GlnD enzyme. Under nitrogen starvation, they are found uridylylated (18). Conversely, upon an increase in nitrogen availability, intracellular L-glutamine levels rise, switching the bifunctional GlnD enzyme to remove the uridine 5′-monophosphate (UMP) from the GlnB and GlnK Y51 (19, 20). Hence, the presence of UMP attached to the GlnK and GlnB Y51 acts as a proxy of nitrogen deficiency (18, 21).

In addition to the ability to indirectly sense L-glutamine, PII proteins also sense the levels of 2-oxoglutarate (2-OG), ATP, and ADP (8, 22, 23, 24). The 2-OG levels act as a signal of the carbon to nitrogen ratio, whereas the ATP:ADP ratio acts as a proxy of availability of cellular energy (25, 26). Three nucleotide-binding sites are located in the lateral clefts between each PII monomer; these sites can be occupied competitively by ATP or ADP (27). Three 2-OG binding sites are formed in the lateral clefts between each PII monomer, formed as a consequence of the occupation of the nucleotide binding site by Mg.ATP (28, 29). As such, the binding of 2-OG and ATP shows positive cooperativity, whereas the binding of ADP and 2-OG shows negative cooperativity (25).

The ability of PII proteins to sense energy (ATP:ADP ratio), carbon (2-OG), and nitrogen levels (L-glutamine) was capitalized by nature in such a way that PII proteins act as a metabolic hub to regulate the activity of a vast range of other proteins by means of protein–protein interaction (5, 30, 31). The physical interaction between PII and its target proteins is regulated by the structural changes induced in the PII structure upon binding or dissociation of the allosteric effectors (ATP, ADP, and 2-OG) and by the reversible uridylylation (8, 21, 29, 32, 33). Recent studies indicate that PII proteins play a broad regulatory role in bacterial metabolism (3). In addition to the well-studied function as regulator of nitrogen assimilatory pathways, recent data from *E. coli* and from other proteobacteria indicate that PII can control nitrogen degradation pathways (34, 35), biosynthesis of NAD^+^ (36), fatty acid production (37, 38) and c-di-GMP levels (39).

This study was set to identify novel PII binding partners in *E. coli.* We used the PII proteins as bait to identify the ribonucleotide monophosphatase UmpH (previously named NagD) as a specific target of the GlnK protein. The UmpH enzyme is a member of the haloacid dehalogenase superfamily, and the structure of its monomer has been solved (40). The UmpH structure comprises a conserved α/β core domain, which carries the catalytic site. The UmpH also contains a cap domain whose function is believed to confer the specificity of the substrate among members of the dehalogenase family (40).

*In vitro* analysis revealed that UmpH acts as a phosphatase toward a wide range of substrates with preference to monophosphate nucleotides. Among the substrates evaluated, UMP seemed to be the most relevant at physiological concentrations (40). Indeed, metabolomic and genetic analyses support that UmpH uses UMP as substrate *in vivo* (41).

Here, we show that UmpH interacts with GlnK *in vitro*. The interaction is regulated according to levels of ATP, ADP, 2-OG, and by the status of GlnK uridylylation. The formation of the GlnK–UmpH complex affects the kinetic properties of the UmpH enzyme resulting in reduced catalytic efficiency by augmenting the enzyme *K*_*M*_ for UMP. We propose a model where GlnK acts as a switch to downregulate UmpH upon an ammonium shock reducing UMP degradation when nitrogen becomes available.

## Results

### Identification of UmpH as novel GlnK interacting partner in *E. coli*

To identify novel PII protein targets, N-terminal His-tagged GlnK or GlnB proteins were immobilized as baits onto Ni^2+^ columns, which were incubated with cell-free extracts of *E. coli ΔglnBglnK* in the presence of Mg.ATP. After extensive washes, proteins that were retained by His-PII proteins were selectively eluted in buffer containing 1 mM of Mg.ATP and 1.5 mM of 2-OG. The rational of this approach is that PII proteins adopt a different structure upon 2-OG binding, thereby altering the stability of PII–target protein complexes that were eventually formed in the presence of Mg.ATP (29).

**Figure 1:**
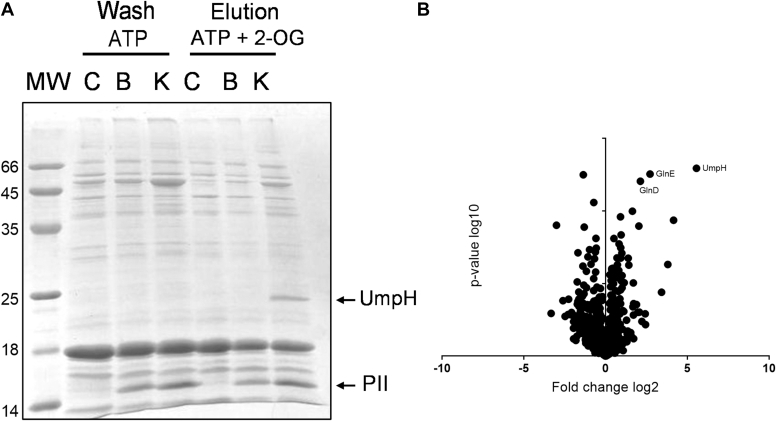
**Ligand fishing analysis.***A*, comparison of the SDS-PAGE protein profiles of the last Mg.ATP wash fraction with the profile obtained after elution with Mg.ATP and 2-OG combined from the different ligand-fishing columns. Empty control column (C), His-GlnB bait column (B), and His-GlnK bait column (K). MW indicate molecular weight markers (kDa). The UmpH and PII bands are indicated by *arrows*. *B*, the proteins eluted from the His-GlnK column after the Mg.ATP + 2-OG treatment were compared with those eluted from the control column using label-free LC/MS/MS analysis. Volcano plots were prepared using the significance of enrichment (*p* value log 10) *versus* the enrichment fold (fold change log 2). The most significant enriched proteins in the His-GlnK column in comparison to the control column are indicated by their respective names GlnE, GlnD, and UmpH. 2-OG, 2-oxoglutarate.

Comparison of the SDS-PAGE protein profiles of the last Mg.ATP wash fraction with the profile obtained with MgATP and 2-OG revealed a band of approximately 30 kDa eluting specifically in the presence of 2-OG from the GlnK column (Fig. 1*A*). This band was present in neither the control nor the column where GlnB was used as bait (Fig. 1*A*). The band indicated by an *arrow* in Figure 1 was excised from the gel, digested with trypsin, and analyzed by MALDI-TOF mass spectrometry. Peptide mass fingerprint searches identified that band as NagD, currently named as UmpH (42% sequence protein coverage). This identification was confirmed by MS/MS ion search of the ion of *m/z* 2514, which matched one of the UmpH peptides (ion score 83 with scores >40 being significant at *p* < 0.05).

The proteins eluted from the His-GlnK column after the 2-OG treatment were compared to those eluted from the control column using label-free LC/MS/MS analysis. Volcano plots were performed plotting the significance of enrichment (log 10 *p* value) *versus* the enrichment fold (log twofold change). The graph indicates that UmpH stands out as the major protein enriched in the 2-OG eluate from the column where His-GlnK was used as a bait (Fig. 1*B*). Two other well-characterized GlnK targets, GlnD and GlnE, were also among the most enriched proteins along with UmpH, thereby validating the biological significance of the assay (Fig. 1*B*).

### Characterization of the UmpH–GlnK complex

**Figure 2:**
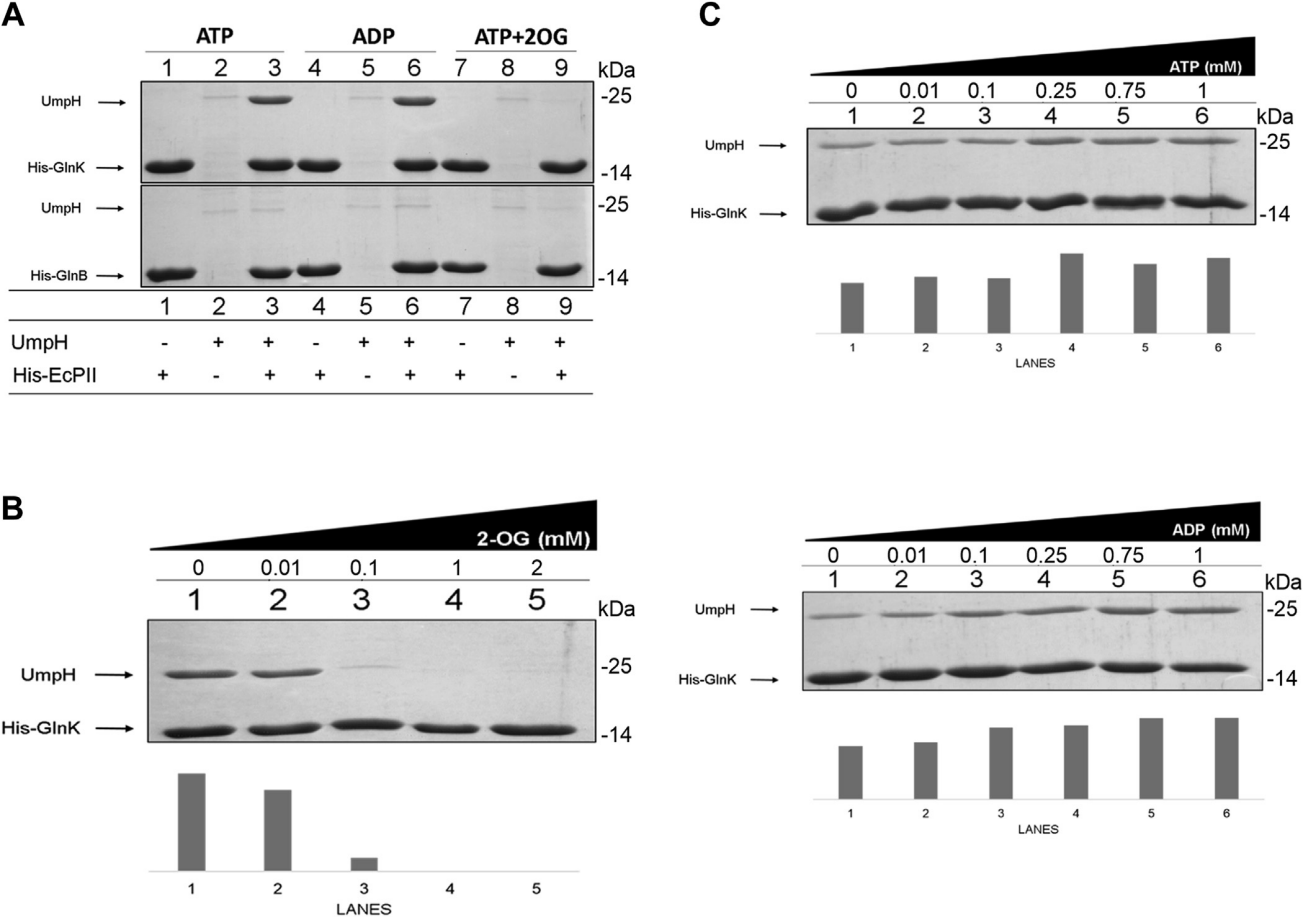
***In vitro* complex formation between UmpH and PII.***A*, pull-down was performed in the presence of MgCl_2_ (5 mM) and the indicated effectors ATP, ADP, and 2-OG at 1 mM. The binding reactions contained His-PII (20 μg) and UmpH (40 μg). Proteins eluted from the Ni^2+^ magnetic beads were analyzed by SDS-PAGE. *B*, pull-down was performed under fixed concentration of ATP (1 mM) and MgCl_2_ (5 mM) and increasing concentrations of 2-OG as indicated. *C*, pull-down reactions were performed under fixed concentration of MgCl_2_ (5 mM) and increasing concentrations of ATP or ADP as indicated. The *bars* in *B* and *C* indicate the densitometry analysis of the band corresponding to UmpH in each lane. 2-OG, 2-oxoglutarate.

To confirm the specificity of the identified UmpH–GlnK protein interaction, untagged recombinant UmpH was purified to homogeneity and challenged for interaction using His-GlnK or His-GlnB as bait by coprecipitation using Ni^2+^ magnetic beads under different conditions. The data shown in Figure 2*A* confirmed that UmpH coelutes with GlnK but not with GlnB. The UmpH–GlnK protein interaction could be detected in the presence of ADP and ATP but not when ATP and 2-OG were combined (Fig. 2*A*).

To further investigate the effect of 2-OG on the interaction, an assay was carried out in the presence of ATP and different concentrations of 2-OG (Fig. 2*B*). In the presence of 0.01 mM of 2-OG, complex formation occurred as in the absence of 2-OG. However, at 0.1 mM of 2-OG, protein interaction decreased significantly, and 1 mM of 2-OG completely prevented GlnK–UmpH interaction. Different concentrations of ADP and ATP were also evaluated. The results show that the UmpH–GlnK interaction can occur without the presence of nucleotides; however, with increasing ATP or ADP concentrations, increasing coprecipitation of UmpH with GlnK was obtained (Fig. 2*C*), suggesting that both nucleotides can stabilize the protein complex.

Complex formation between UmpH and GlnK was also evaluated under different combinations of ADP, ATP, and 2-OG (Fig. S1). The data indicate that 2-OG abrogates complex formation only under a high ATP:ADP ratio. When the ATP:ADP ratio drops while keeping the total ATP + ADP = 1 mM, the ability of 2-OG to inhibit UmpH–GlnK complex formation is reduced (Fig. S1). When only ADP is present, 2-OG could not inhibit complex formation as expected (Fig. S1). These data suggest that not only the 2-OG levels but also the ATP:ADP ratio can affect the interaction between UmpH and GlnK.

In addition to the control exerted by the allosteric effectors ATP, ADP, and 2-OG, the GlnK activity is also modulated by reversible uridylylation. We obtained fully uridylylated GlnK and challenged this preparation to interact with UmpH under different combinations of the allosteric effectors, unmodified GlnK was used in parallel as positive control. The data shown in Fig. S2 indicate that GlnK-UMP_3_ does not interact with UmpH at any combination of effector molecules present.

To obtain structural insights into the selective basis of the interaction between UmpH and GlnK, we performed pull-down assays with orthologous PII, GlnZ and GlnB, from the α-proteobacterium *Azospirillum brasiliense* (GlnZAb and GlnBAb). Interestingly, UmpH was able to interact with both *Azospirillum brasilense* PII, GlnZAb and GlnBAb (Fig. S3*A*). The high sequence and structural similarities among the different PII suggest that the positions that are unique to the GlnBEc sequence (the only PII that did not interact with UmpH) could form the UmpH binding site.

An alignment of the GlnKEc, GlnBEc, GlnZAb, and GlnBAb sequences showed that residues unique to GlnBEc are mostly concentrated between residues 69 and 82 (Fig. S4*A*). Among these candidate positions, surface-exposed residues were mapped to the GlnKEc structure (Fig. S4*B*). This analysis suggests that, differently from most of the PII–target complex known to date, the lateral face of the PII monomers could act as the UmpH binding site (Fig. S4*B*). As a proof of concept, UmpH was able to interact with a GlnKAb variant carrying a deletion on the T-loop region (GlnZΔ42–54) (Fig. S3*B*). However, the GlnZΔ42–54–UmpH complex was not negatively regulated by 2-OG (Fig. S3*B*). These data support that even though the T-loop is not required for the PII–UmpH interaction, it plays a role in the response of the PII–UmpH complex to the 2-OG levels (Fig. S3*B*).

**Figure 3:**
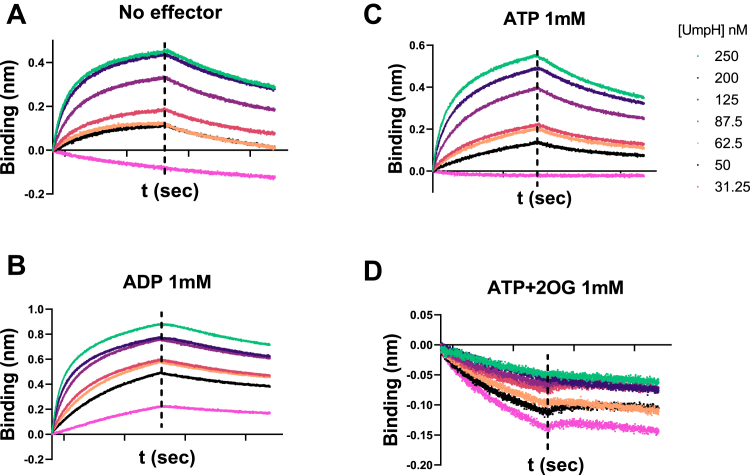
**Biolayer interferometry (BLI) assays of the UmpH GlnK complex.** The purified His-GlnK was mobilized onto a Ni–NTA biosensor, and the tip was dipped into a solution containing the indicated concentrations of UmpH to record the association curve. The sensor was dipped in buffer without UmpH to monitor complex dissociation, indicted as the time interval after the *vertical dashed lines* on each graph. Data were recorded in duplicates and analyzed with the Octet Data Analysis software (Fortébio). The buffers contained no effector (*A*), 1 mM ADP (*B*), 1 mM ATP (*C*), or 1 mM ATP + 2-OG (*D*). 2-OG, 2-oxoglutarate.

The kinetic parameters of the interaction between UmpH and GlnK were assessed using biolayer interferometry. His-tagged GlnK was immobilized onto a nickel sensor and challenged with UmpH under different effector conditions. The formation of the UmpH–GlnK complex could be detected in the absence of allosteric effector and in the presence of ADP or ATP but not when ATP and 2-OG was combined (Fig. 3). The affinity of the UmpH–GlnK complex was high in the presence of ADP with an equilibrium dissociation constant *K*_*d*_ = 7.8 nM ± 0.05 (Fig. 3). The binding affinity was 37.3 nM ± 0.21 and 50.6 nM ± 0.77, in the presence of ATP or without effectors, respectively (Fig. 3).

### GlnK inhibits the phosphatase activity of UmpH

**Figure 4:**
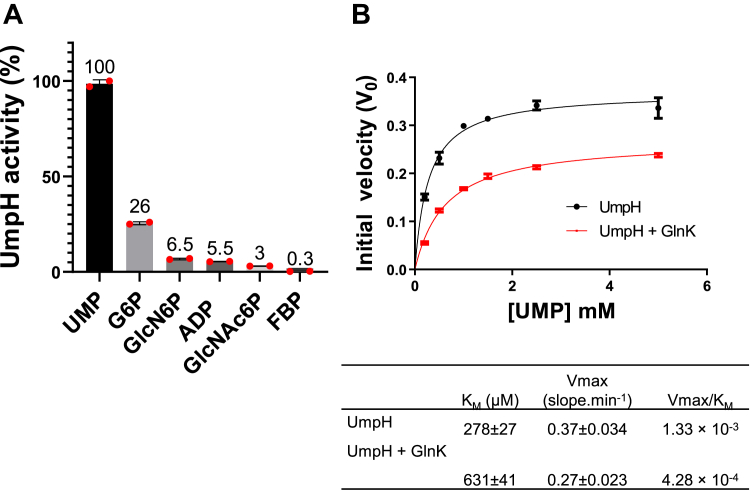
***In vitro* activity of UmpH.***A*, different phosphosubstrates were tested as UmpH substrate at 1 mM. The activity of UmpH using the different substrates was plotted as the percentage of the activity using UMP as a reference. Error bars and the data points are indicated. *B*, kinetic analysis of UmpH. Initial velocities (V_0_) were measured using 0.56 μM of UmpH monomer and different concentrations of UMP. The *red line* was obtained in the presence of 2 μM GlnK (trimer concentration). Reactions were performed in the absence of GlnK effectors. The kinetic parameters are indicated in the table. FBP, fructose 1,6-biphosphate; G6P, glucose-6-phosphate; GlcN6P, glucosamine 6-phosphate; GlcNAc6P, *N*-acetylglucosamine 6-phosphate; UMP, uridine 5-monophosphate.

After confirming the UmpH–GlnK interaction *in vitro*, we hypothesized that GlnK could act to control the enzymatic activity of UmpH. Hence, the activity of UmpH was determined *in vitro* by continuously measuring the phosphate release. Previous studies indicated that even though UmpH can act as a phosphatase over different substrates, both *in vitro* and *in vivo* analyses support that UMP is the physiological relevant substrate (40, 41). Indeed, UmpH was more active with UMP as substrate as compared to phosphosugars, such as glucose-6-phosphate, glucosamine 6-phosphate, *N*-acetylglucosamine (NAG) 6-phosphate, and fructose 1,6-biphosphate when these substrates were at 1 mM (Fig. 4*A*). ATP and ADP were also tested as potential substrates for UmpH activity since they were used in some experiments as GlnK effectors. While UmpH showed minor activity using ADP as substrate (Fig. 4*A*), no UmpH activity could be detected using 1 mM ATP (data not shown).

Kinetic parameters were obtained assaying UmpH activity under different UMP concentrations in the absence and presence of GlnK. The UmpH enzyme showed a typical hyperbolic Vo *versus* UMP concentration curve. Fitting the experimental data into the Michaelis–Menten equation resulted in an *K*_*M*_ = 278 ± 27 μM, which is close to the value determined in a previous study of 160 ± 38 μM (40). The presence of GlnK altered the kinetic parameters of the UmpH reaction, the *K*_*M*_ for UMP increased 2.3x, reaching 631 ± 41 μM, whereas the *V*_max_ decreased about 30% (Fig. 4*B*). The overall UmpH catalytic efficiency (*V*_max_/*K*_*M*_) decreased 68% in the presence of GlnK (Fig. 4*B*). These data support that, when complexed to GlnK, UmpH altered its kinetic parameters reducing its affinity for UMP.

**Figure 5:**
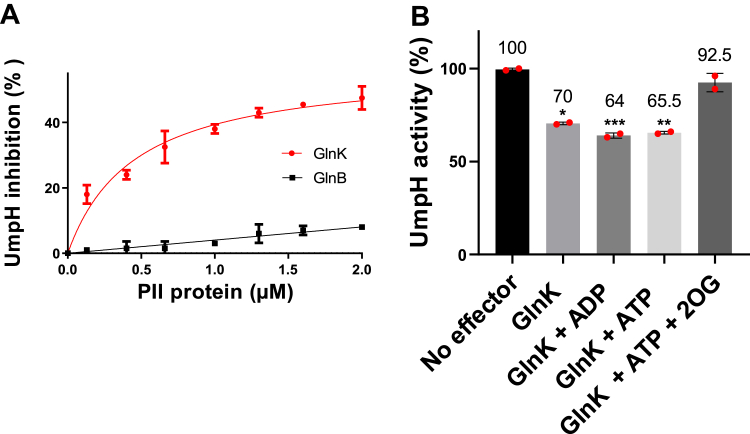
**Inhibition of UmpH enzymatic activity by GlnK.***A*, the reactions were performed using 0.5 mM of UMP as UmpH substrate and 0.1 mM ADP as GlnK effector. Reactions contained 0.56 μM of UmpH and the indicated trimer concentrations of GlnK (*red line*) or GlnB (*black line*). The data are represented as a percentage of inhibition using a reaction without PII as reference. *B*, the reactions were performed using 0.5 mM UMP as UmpH substrate and in the presence of 1 mM of the indicated GlnK effectors. Reactions contained 0.3 μg of UmpH and 5 μg of GlnK (if indicated). The reaction containing only UmpH (*black bar*) was set as a reference of 100% activity. Mean values ± SD were compared using one-way ANOVA. Significant values of *p* = 0.0003, 0.0002, and 0.0001 are indicated by ∗, ∗∗, and ∗∗∗, respectively. Errors bars and the data points are indicated.

The negative effect of GlnK over UmpH activity was dose dependent. Increasing the amount of GlnK augmented the inhibition of UmpH activity, with a maximum inhibition of UmpH activity of approximately 50% being reached at a molar ratio of UmpH monomer:GlnK trimer of 0.56:2 μM (Fig. 5*A*). Addition of GlnB, which cannot interact with UmpH (Fig. 2*A*), produced negligible effect over UmpH activity (Fig. 5*A*).

The UmpH activity was measured in the presence of GlnK and different PII allosteric effectors (all at 1 mM) using UMP at 0.5 mM, which is close to the *K*_*M*_ of the enzyme. GlnK was able to inhibit UmpH activity (*p* < 0.05) only under conditions where the GlnK–UmpH complex formation was observed: in the presence of ADP or ATP, or in the absence of effectors, but not when ATP and 2-OG were combined (Fig. 5*B*). This assay was also performed at lower concentrations of the GlnK effectors (0.1 mM), the same profile observed (Fig. S5). These data support that UmpH activity is negatively regulated by interaction with GlnK.

## Discussion

In prokaryotes, nucleotides not only act as important energy metabolites in various cellular processes but also as building blocks for nucleic acid RNA and DNA production (42). Conversely, under starvation conditions, nucleotides from the environment or from degrading nucleic acids can be fed into catabolic pathways (43). Therefore, nucleotide biosynthetic and degradation pathways must be tuned not only to avoid futile cycles but also to pace the flow-through in each of these pathways accordingly to the availability of nutrients, such as carbon, nitrogen, and energy sources (44). Fine tuning nucleotide metabolism is likely to be important for fitness in bacteria, which experiences feast and famine cycles such as *E. coli* (45).

Despite the wealth of data regarding the regulation of nucleotide biosynthetic pathways, the regulation of nucleotide degradation pathways came into focus of research only in recent years. Regulation of nucleotide degradation may occur at the transcriptional level. For instance, in the uracil degradation pathway, the RutR repressor dissociates from its operator sites in the presence of uracil allowing the transcription of uracil-degrading genes (46, 47). Analysis of absolute metabolite levels in *E. coli* suggests that nucleotide-degrading enzymes are regulated by substrate availability as the *K*_*M*_ of the degrading enzymes typically feel short the substrate concentrations during steady state growth conditions (48). This seems to be the case of UmpH, which exhibits a *K*_*M*_ for UMP of 278 μM in contrast to the determined UMP intracellular concentration of 52 μM during steady state grow conditions (41).

**Figure 6:**
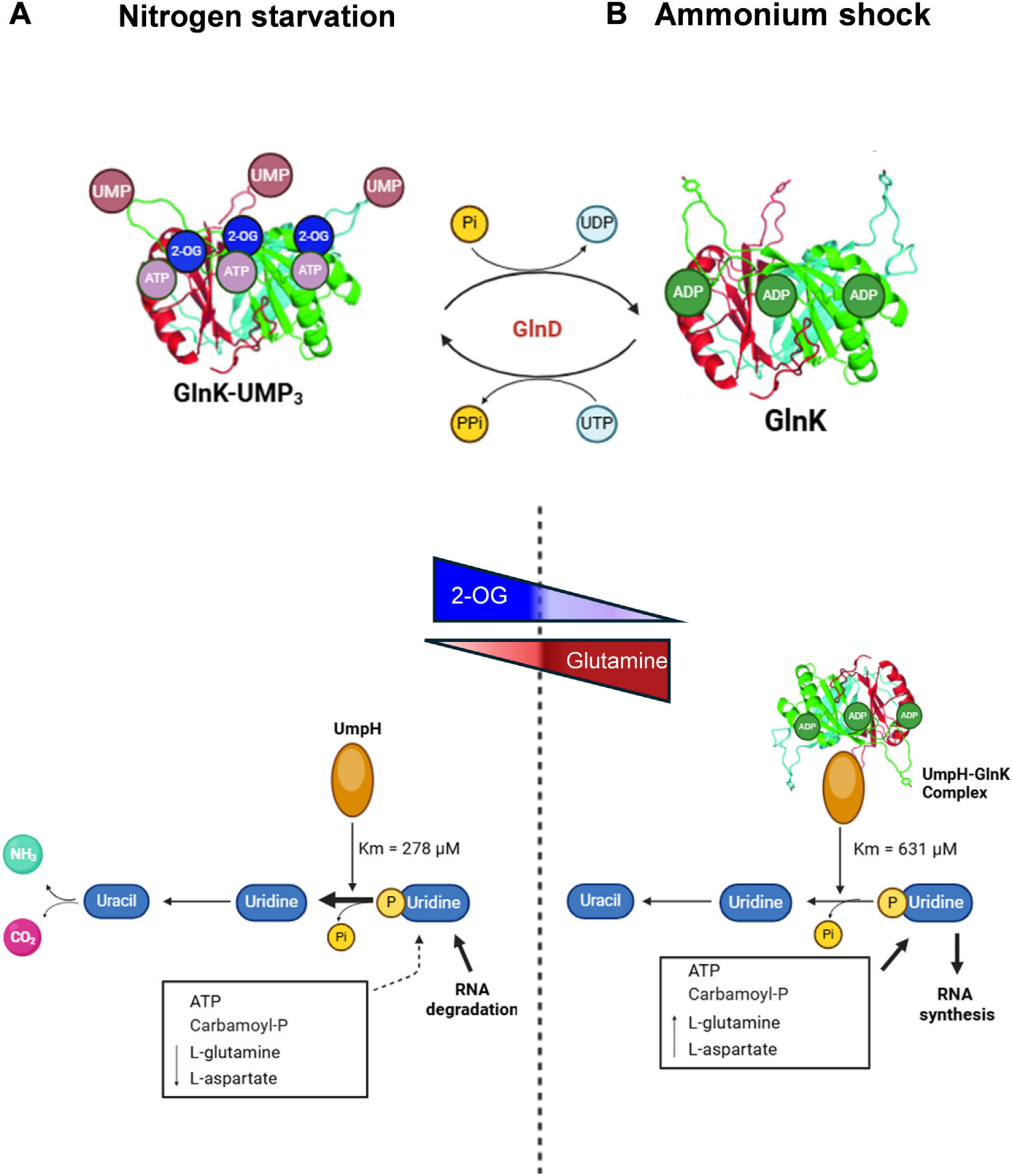
**Regulatory model of UmpH activity by GlnK.***A*, under nitrogen starvation, GlnK is fully uridylylated and cannot interact with UmpH, which is active. Degradation of UMP can fill the *rut* uridine catabolic pathway, which is induced under nitrogen starvation. The degradation of unused RNAs could act as a source of UMP for UmpH activity. *B*, upon an ammonium shock, the L-glutamine levels rise and the 2-OG levels drop. GlnK is rapidly deuridylated and bound to ADP. This condition promotes the interaction between GlnK and UmpH reducing the enzyme activity and UMP degradation. At the same time, increased L-glutamine and L-aspartate will favor UMP biosynthesis. This regulatory mechanism could help the cells to rapidly switch from a catabolic to an anabolic state in response to ammonium availability in the medium. 2-OG, 2-oxoglutarate.

Here, we used ligand fishing assays to identify UmpH as a novel target of PII signaling protein GlnK. The interaction is specific for GlnK, as no interaction could be detected with the paralog protein GlnB. As GlnK is induced under nitrogen starvation, it is expected that the UmpH–GlnK interaction could play a role during nitrogen starvation and/or during the transition from N-starvation to N-sufficiency. Biochemical analysis showed that the GlnK–UmpH interaction is abrogated when GlnK is uridylylated or bond to Mg.ATP and 2-OG (Figs. 2*A*, 3*D*, and S2). When nitrogen-starved cells encounter nitrogen sources, GlnK gets rapidly deuridylylated and interacts avidly with UmpH. Therefore, the GlnK–UmpH complex is expected to form after an ammonium shock, conditions where the intracellular L-glutamine rises favoring GlnK deuridylylation, and the 2-OG levels drop favoring the allosteric binding of ADP to GlnK (Fig. 6*B*). The formation of the UmpH–GlnK complex reduces the UmpH *K*_*M*_ for UMP, and thus UmpH activity after the transition from N-starvation to N-sufficiency conditions (Fig. 6*B*).

Nitrogen starvation reduces the availability of L-glutamine and L-aspartate (49, 50), which are precursors used for both protein and nucleotide biosynthesis, including UMP (Fig. 6*A*). During nitrogen starvation, the lack of amino acids triggers the stringent response reducing stable RNA (rRNA and tRNA) production (51). At the same time, starvation promotes RNA degradation, which is likely to increase the levels of UMP to feed UmpH activity (52). The uridine nucleoside produced by UmpH can be recycled into ammonium through the *rut* pathway (41, 46, 53). It has been suggested that the ammonium derived from different catabolic pathways, including *rut*, may act as a nitrogen source to maintain a minimal rate of protein biosynthesis under N-starvation (Fig. 6*A*) (54, 55).

When nitrogen-starved cells find ammonium in the external medium, the cells should rapidly switch from a catabolic to an anabolic state. The increase in L-glutamine and L-aspartate is likely to enhance UMP biosynthesis (Fig. 6*B*). However, instead of feeding the *rut* catabolic pathway, the nucleotide should now fill biosynthetic routes such as RNA biosynthesis (Fig. 6*B*). The formation of the UmpH–GlnK complex under this condition would reduce the affinity of UmpH for UMP, acting as a valve to switch the fate of UMP from degradation to biosynthesis (Fig. 6*B*).

Quite remarkably, the *rut* pathway is also under the control of PII protein signaling (56). The *rut* genes are induced under N-starvation by the action of a sigma 54 promoter activated by NtrC (54, 57). Hence, PII proteins participate in the control of the *rut* catabolic pathway at three levels: (1) regulating the availability of the initial substrate uridine by controlling UmpH activity; (2) regulating the availability of uridine, which controls the RutR repressor; and (3) regulating *rut* gene expression by controlling the activity of NtrC.

In summary, here we identified UmpH as a novel target of the GlnK signaling protein in *E. coli*. We suggest a model where GlnK will interact with UmpH during the transition from N-starvation to N-sufficient conditions, helping the cells to rapidly reprogram its metabolism from a catabolic to an anabolic state to avoid futile cycling of key nutrients.

## Experimental procedures

### PII ligand fishing affinity chromatography

The *E. coli* PII proteins, GlnB or GlnK, were expressed using *E. coli* BL21(DE3) carrying the plasmids pTRPETHisGlnB or pTRPETHisGlnK, respectively. These plasmids are based on pET28a and were described previously (38, 58). Cells were cultured in 300 ml of LB medium containing kanamycin 100 μg.ml^−1^ to an absorbance at 600 nm of 0.5, 0.5 mM IPTG was added, and the culture incubated for 3 h at 37 °C under vigorous shaking. Cells collected by centrifugation, resuspended in 10 ml of buffer A (50 mM Tris–HCl [pH 8]; 0.1 M KCl; 20 mM imidazole), and sonicated on an ice bath. After centrifugation at 20,000*g* for 20 min at room temperature, the soluble fraction was recovered and loaded onto a Protino 1000 Ni-IDA column (Macherey–Nagel). The columns were washed using 15 ml of buffer A containing imidazole 60 mM to remove loosely bound proteins and keep the bait proteins His-GlnB or His-GlnK on two separate columns.

The prey proteins were obtained from *E. coli* FT8000 *ΔglnBglnK* (59). About 300 ml of cells were cultured on LB medium to an absorbance of 0.5 at 600 nm. Cells were collected by centrifugation, resuspended in 10 ml of buffer B (50 mM Tris–HCl [pH 8]; 0.1 M KCl; 20 mM imidazole; 5 mM MgCl_2_), and sonicated on an ice bath. After centrifugation at 20,000*g* for 20 min at room temperature, the soluble fraction was recovered and mixed with ATP to 1 mM final concentration. The two prey columns prepared as described in the previous paragraph (containing mobilized His-GlnB or His-GlnK) along with an empty control Protino 1000 Ni-IDA column (Macherey–Nagel) were connected in series (in this order: control, His-GlnB, and His-GlnK). Columns were washed with 10 ml of buffer C (buffer B containing 1 mM ATP) and loaded with the cell extract form *E. coli* FT8000 *ΔglnBglnK.* The columns were separated and individually washed with 12 ml of buffer C. The final 2 ml was collected to be used as flowthrough background controls. The columns were eluted with 2 ml of buffer D (buffer C containing 1.5 mM 2-OG); this final fraction was collected to identify proteins that were specifically eluted by 2-OG in the presence of PII proteins. The recovered fractions were analyzed by SDS-PAGE or by label-free quantitative LC–MS/MS as described previously (60).

### In-gel protein digestion and mass spectrometry analysis

Protein bands excised from Coomassie-stained SDS-PAGE gels were subjected to in-gel digestion with sequencing-grade trypsin as described (61). MALDI-TOF was performed mixing the hydrolyte sample with a saturated solution of α-cyano-4-hydroxycinnamic acid dissolved in 50% acetonitrile v/v and 0.1% TFA v/v. This mixture was spotted onto the MALDI target plate and allowed to dry. Mass spectra were acquired using a MALDI-TOF/TOF Autoflex II spectrometer (Bruker Daltonics). Raw data were converted to a monoisotopic peak list using the FlexAnalysis 3.0 software (Bruker Daltonics). Database search was performed using the online Mascot server (https://www.matrixscience.com/), the *E. coli* database, and error tolerance of 100 ppm for PMF search, and for parent ion MS/MS search; the MS/MS fragment tolerance error was set to 0.3 Da.

### Label-free LC/MS/MS proteomics

Proteins enriched in the fraction eluted with 2-OG from the His-tagged GlnK Ni^2+^ column were analyzed by label-free LC/MS/MS. Briefly, aliquots of 15 μg of the GlnK affinity column and from the respective control column were suspended in 50 μl of 100 mM ammonium bicarbonate (pH 8.0). Proteins were reduced with 1 mM DTT for 45 min. Modified trypsin (Promega) was added to a 1:50 ratio and incubated overnight at 37 °C. Protein digestion was quenched with TFA 3% (v/v), peptides were extracted with C18 STAGE-TIPs, and subjected to technical triplicate LC/MS/MS runs. Samples were analyzed in a QExactive Orbitrap (Thermo Scientific), and the data were processed using MaxQuant, version 1.5.2.8 (62). Search parameters were trypsin with no Pro restriction, mass deviation of 20 ppm and 6 ppm for first and main search, respectively, and oxidation of Met as variable modification. Proteins were identified using an *E. coli* protein database downloaded from UniProt. Statistical analysis was performed using MaxQuant—Perseus package version 1.5.0.30, and statistically significant differences were assigned using a one-way ANOVA test with *p* value threshold of 0.05 and Benjamin–Hochberg-based false discovery rate correction. Protein abundance obtained after elution with 2-OG from the GlnK affinity column were compared to protein abundance from an empty control column. Proteins enriched in the GlnK column were identified by volcano plots considering the log of *p* value *versus* log2 of fold change.

### Plasmids used for protein expression

The amino acid sequence of the UmpH (NagD) protein was retrieved from UniProt (P0AF24.1). The *umpH* gene was synthetized and cloned into pET29a by General Biosystems. Plasmids, pTRPETHisGlnB and pTRPETHisGlnK, expressing GlnB and GlnK proteins with a histidine tag in the N-terminal region have been described previously (38, 58). The pDOP1 plasmid was used to express the *E. coli* GlnD (63). Plasmids pMSA3 and pLMA-MLV1 were used to express GlnZ and GlnB from *A. brasiliense* with a histidine tag at N-terminal region, respectively (64, 65). The plasmid pMSA4ΔloopT expressing the version of GlnZ containing a deletion on the T-loop (GlnZΔ42–54) was used to generate an N-terminal His-tagged fused version, by subcloning GlnZ NdeI and BamHI fragments of pMSA4ΔloopT into the NdeI and BamHI sites of pET28a (36). The resulting plasmid was named pGAHisGlnZΔloop and was used to express His-GlnZΔloop.

### Protein purification

Untagged UmpH and His-tagged GlnB and GlnK were expressed in *E. coli* BL21 (λDE3) carrying the respective expression plasmid. Cells were cultivated in 300 ml of LB medium containing 100 μg.ml^−1^ kanamycin at 37 °C with continuous 120 rpm shaking. When cells reached an absorbance at 600 nm between 0.3 and 0.4, the cultured temperature was set to 16 °C before the addition of 0.3 mM IPTG. The culture was further incubated overnight at 16 °C with continuous 120 rpm shaking. During GlnB and GlnK protein expression, 40 mM ammonium chloride was added to the culture before the addition of IPTG to avoid protein uridylylation. For GlnD expression, the antibiotic used was 100 μg.ml^−1^ ampicillin, and the protein expression was achieved after the cells reached an absorbance at 600 nm between 0.3 and 0.4 by changing the shaker temperature from 37 °C to 42 °C. Cells were incubated for 3 h at 42 °C before being collected by centrifugation.

To purify UmpH, cells were resuspended in 25 ml of sonication buffer (50 mM Hepes [pH 7.4], 10 mM MgCl_2_, and 5 mM DTT) and disrupted by sonication on ice. Cell extracts were clarified by centrifugation (20,000*g* for 15 min at 4 °C), and 20% of ammonium sulfate was added to the supernatant, which was kept on ice for 20 min. After another round of centrifugation, the supernatant was discarded and the pellet was resuspended in 4 ml of resuspension buffer (50 mM Hepes [pH 7.4], 10 mM MgCl_2_, and 2 mM DTT). The supernatant was dialyzed overnight at 4 °C in 1 l of dialysis buffer (50 mM Hepes [pH 7.4], 10 mM MgCl_2_, 2 mM DTT, and 50% glycerol). After the dialysis, 100 mM of NAG was added as NAG was shown to stabilize the UmpH activity and reduce aggregation (40). The protein preparation was polished using gel filtration chromatography. Samples were separated using a Superdex 200 HiLoad 26/60 (Cytiva), which were equilibrated with two volumes of buffer (50 mM Hepes [pH 7.4], 100 mM NaCl, 10 mM MgCl_2_, 2 mM DTT, and 20 mM NAG). The elution of UmpH was monitored by SDS-PAGE.

The purification of His-GlnB, His-GlnK, and GlnD was performed as described previously (58, 66). His-GlnZ, His-GlnB, and His-GlnZΔloop from *A. brasiliense* were purified as described previously (64, 65, 66). Fully uridylylated GlnB and GlnK proteins were obtained as described previously, and the state of full uridylylation was confirmed by native PAGE analysis (67). All proteins used in this study were quantified using the Bradford assay (Sigma) and kept at −80 °C until use.

### *In vitro protein* complex analysis

*In vitro* complex formation was performed using Magne His nickel magnetic beads (Promega) as described previously (65). All reactions were conducted in buffer containing 50 mM Tris–HCl (pH 8.0), 0.1 M NaCl, 5 mM MgCl_2_, 10% glycerol (v/v), and 20 mM imidazole in the presence or the absence of effectors as indicated in each experiment. Four microliters of the beads were equilibrated by wash with 200 μl of buffer. Binding reactions were performed in 200 μl of buffer by adding 10 μg of His-EcPII or His-AbPII and then 20 μg untagged UmpH. The proteins were mixed at room temperature for 5 min. The beads were washed three times with 200 μl of buffer, and samples eluted with SDS-PAGE sample buffer and analyzed by SDS-PAGE. Gels were stained with Coomassie blue, and gel band densitometry analyses were performed using Gel Analyzer 19.1 (www.gelanalyzer.com).

### UmpH phosphatase activity assays

The UmpH phosphatase activity was continuously measured using the EnzChek Pyrophosphate Assay Kit (Thermo Fisher; E6645), without the addition of pyrophosphatase. The assays were performed duplicates in flat-bottom polystyrene plates (OLEN) containing 180 μl of 20 mM Hepes (pH 7.4), 50 mM KCl, 5 mM MgCl_2_, 2 mM NAG, 2 mM DTT, 0.1 mM 2-amino-6-mercapto-7-methyl-purine riboside, 1 U/ml purine nucleoside phosphorylase, and 300 ng UmpH). Reactions were preincubated at 25 °C before the addition of 20 μl of the UmpH substrate indicated in each experiment. Reactions were performed in duplicates and continuously monitored measuring absorbance at 360 nm at 25 °C using a Tecan infinity 200 microplate reader (Tecan). The mean slope of the linear phase of absorbance at 360 nm *versus* time was used to obtain the initial velocity in each condition. Initial velocities were fitted into the Michaelis–Menten equation using GraphPad Prism 7 (GraphPad Software, Inc).

### Biolayer interferometry assays

To obtain the kinetic parameters of the GlnK–UmpH complex, an Octet K2 Biolayer Interferometry System (FortéBIO) was used. The purified His-GlnK and untagged UmpH were diluted in the interaction buffer (20 mM Hepes [pH 7.4], 50 mM KCl, 5 mM MgCl_2_, 2 mM DTT, and 2 mM NAG). The Ni–NTA biosensor was first dipped into a solution containing His-GlnK at 9.7 μg/ml for 100 s until a binding signal of approximately 2 nm was obtained. The sensor was washed in binding buffer and then transferred to the analyte solution containing UmpH at different concentrations for 180 s to record the association curve. Finally, the sensor was dipped into the interaction buffer for 180 s to monitor complex dissociation. These analyses were also carried out in the presence of the PII protein effectors 1 mM ADP, ATP, or ATP plus 2-OG as indicated in each figure. Data were recorded in duplicates and analyzed with the Octet Data Analysis software using Savitzky–Golay filtering. The fitting of the curve was done with a 1:1 (GlnK trimer:UmpH monomer) ligand model. Curves were then plotted in GraphPad Prism 7 software.

## Data availability

Data are available upon request to the corresponding author.

## Supporting information

This article contains supporting information.

## Conflict of interest

The authors declare that they have no conflicts of interest with the contents of this article.
