# Supplementary material for: The activity of the ribonucleotide monophosphatase UmpH is controlled by interaction with the GlnK signaling protein in Escherichia coli
Source: J Biol Chem. 2024 Oct 24;300(12):107931. doi: 10.1016/j.jbc.2024.107931 (PMC11617674; doi:10.1016/j.jbc.2024.107931)
Supplement: Supporting Information [file mmc1.docx]

**Supporting Information**

**The activity of the ribonucleotide monophosphatase UmpH is controlled by interaction with the GlnK signaling protein in *Escherichia coli***

Ana Carolina Aparecida Gonçalves^1^, Tatiana de Mello Damasco Nunes^1^, Erick Parize^2^_,_ Edileusa Cristina Marques Gerhardt^2^, Gustavo Antônio de Souza^3^, Jörg Scholl^4^, Karl Forchhammer^4^ and Luciano Fernandes Huergo^1,2^*

From the: ^1^Setor Litoral, UFPR Matinhos, PR, Brazil ; ^2^Programa de Pós-Graduação em Ciências - Bioquímica, UFPR Curitiba, PR, Brazil; ^3^Dept of Biochemistry, Universidade Federal do Rio Grande do Norte, Natal, RN, Brazil; ^4^Interfakultäres Institut für Mikrobiologie und Infektionsmedizin der Eberhard-Karls Universität Tübingen, Auf der Morgenstelle 28, Tübingen 72076, Germany;

**Supporting Figures**

**
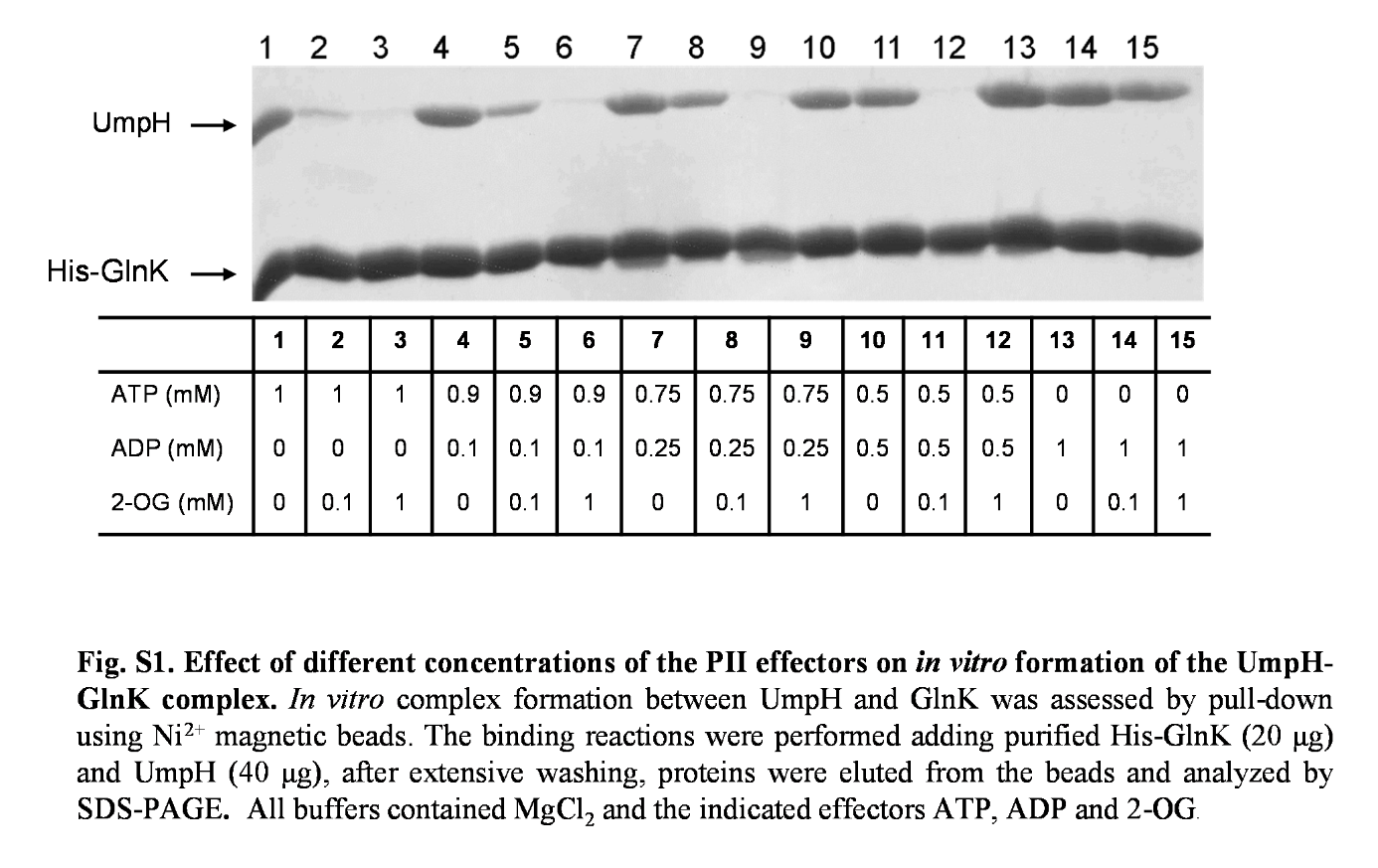
**

**Figure S1. Effect of different concentrations of the effectors on *in vitro* formation of the UmpH-GlnK complex.** *In vitro* complex formation between UmpH and GlnK was assessed by pull-down using Ni^2+^ magnetic beads. The binding reactions were performed adding purified His-GlnK (20 μg) and UmpH (40 μg), after extensive washing, proteins were eluted from the beads and analyzed by SDS-PAGE. All buffers contained MgCl_2_ and the indicated effectors ATP, ADP and 2-OG.


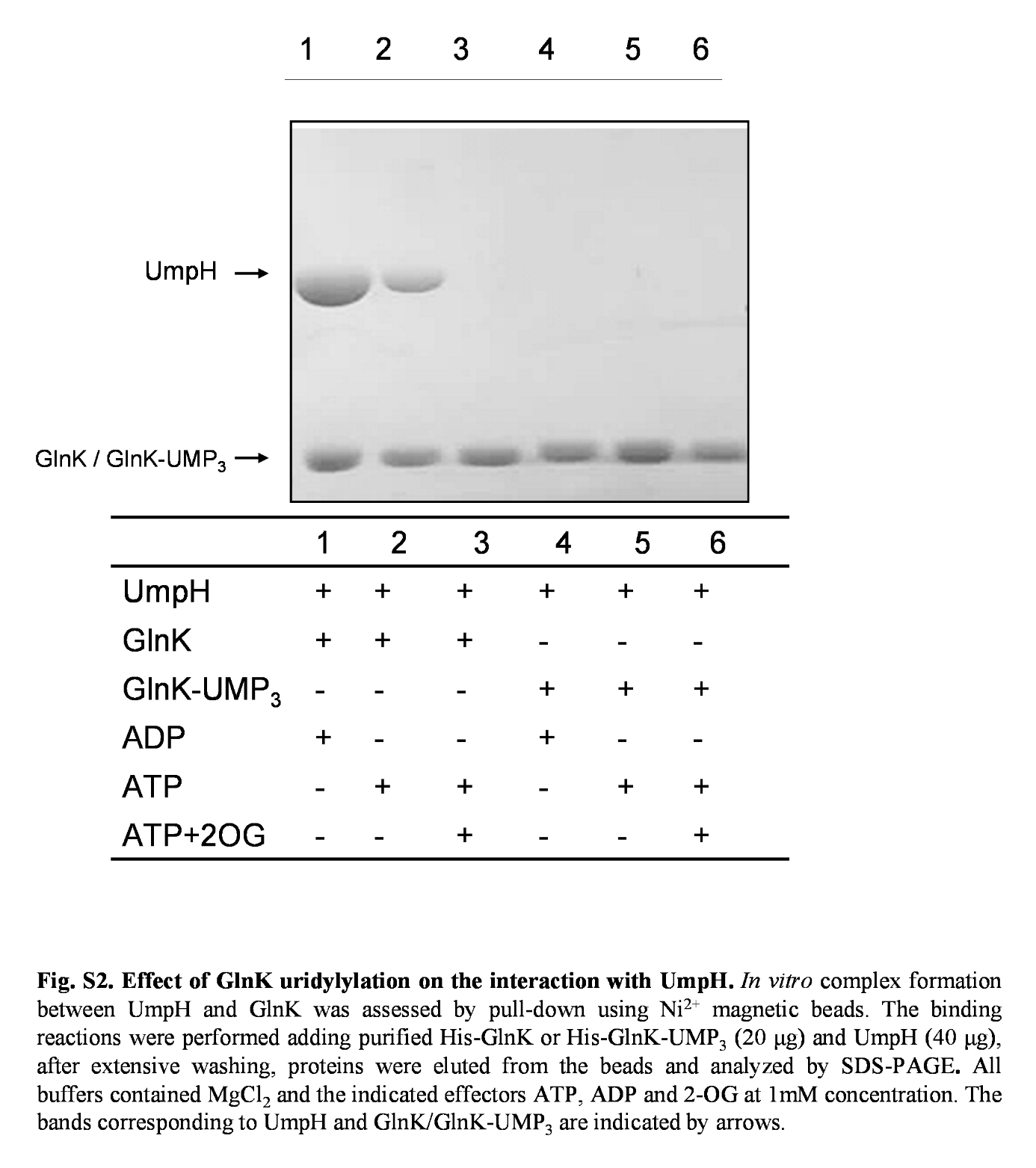


**Figure S2. Effect of GlnK uridylylation on the interaction with UmpH.** *In vitro* complex formation between UmpH and GlnK was assessed by pull-down using Ni^2+^ magnetic beads. The binding reactions were performed adding purified His-GlnK or His-GlnK-UMP_3_ (20 μg) and UmpH (40 μg), after extensive washing, proteins were eluted from the beads and analyzed by SDS-PAGE**.** All buffers contained MgCl_2_ and the indicated effectors ATP, ADP and 2-OG at 1mM concentration. The bands corresponding to UmpH and GlnK/GlnK-UMP_3_ are indicated by arrows.


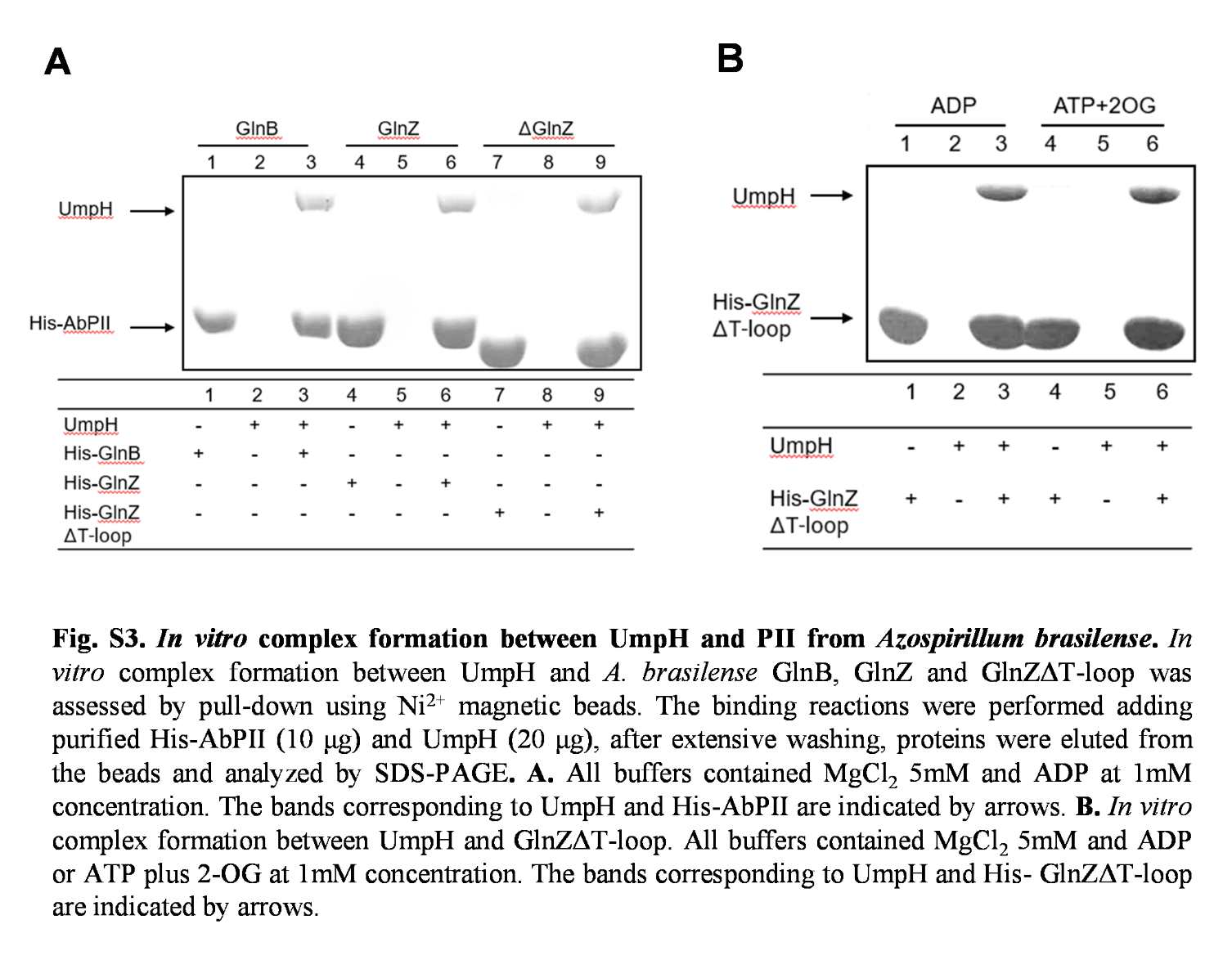


**Fig. S3. *In vitro* complex formation between UmpH and PII from *Azospirillum brasilense*.** *In vitro* complex formation between UmpH and *A. brasilense* GlnB, GlnZ and GlnZΔT-loop was assessed by pull-down using Ni^2+^ magnetic beads. The binding reactions were performed adding purified His-AbPII (10 μg) and UmpH (20 μg), after extensive washing, proteins were eluted from the beads and analyzed by SDS-PAGE. **A.** All buffers contained MgCl_2_ 5mM and ADP at 1mM concentration. The bands corresponding to UmpH and His-AbPII are indicated by arrows. **B.** *In vitro* complex formation between UmpH and GlnZΔT-loop. All buffers contained MgCl_2_ 5mM and ADP or ATP plus 2-OG at 1mM concentration. The bands corresponding to UmpH and His- GlnZΔT-loop are indicated by arrows.


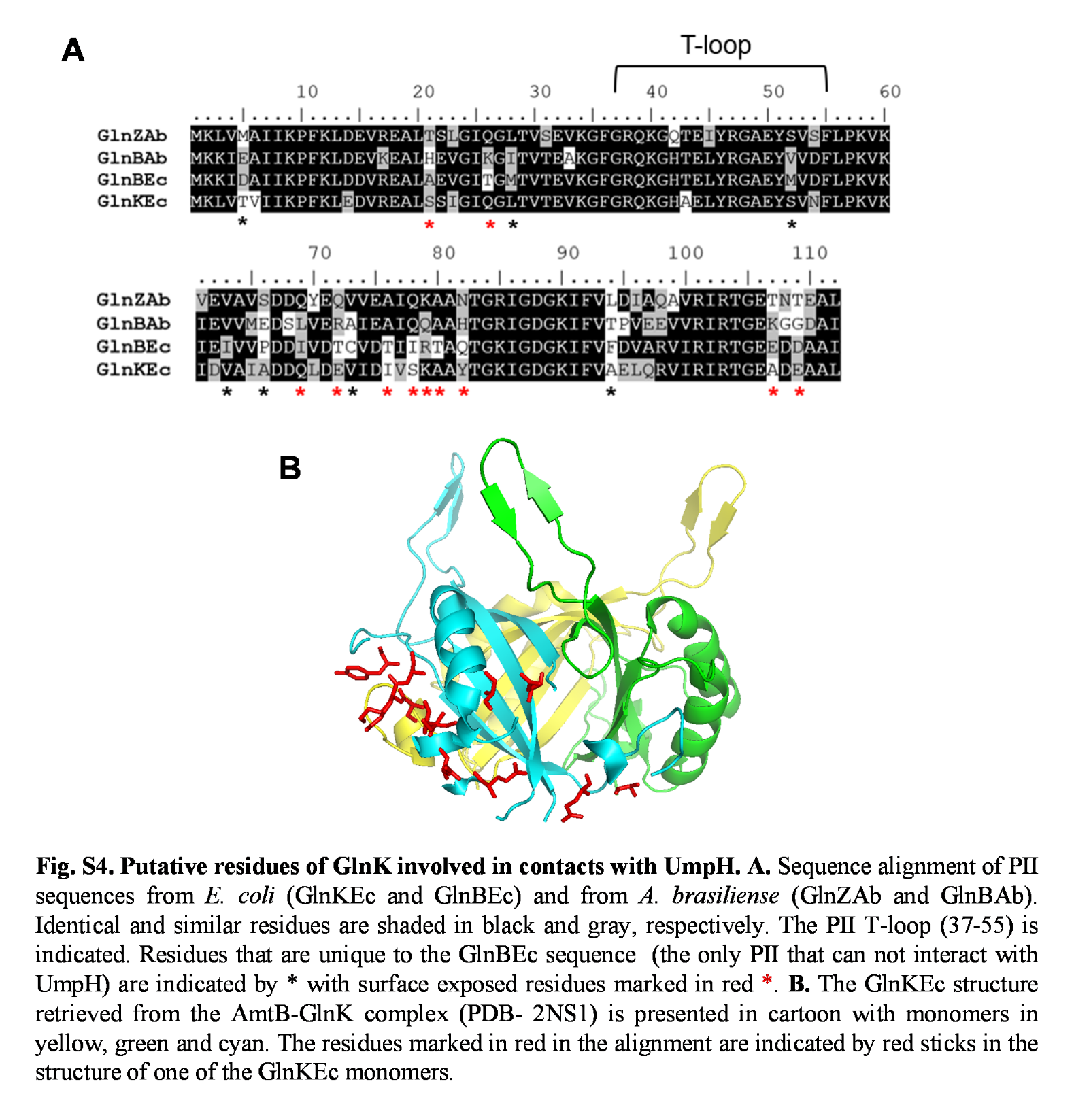


**Fig. S4. Putative residues of GlnK involved in contacts with UmpH.** **A.** Sequence alignment of PII sequences from *E. coli* (GlnKEc and GlnBEc) and from *A. brasiliense* (GlnZAb and GlnBAb). Identical and similar residues are shaded in black and gray, respectively. The PII T-loop (37-55) is indicated. Residues that are unique to the GlnBEc sequence (the only PII that cannot interact with UmpH) are indicated by * with surface exposed residues marked in red *. **B.** The GlnKEc structure retrieved from the AmtB-GlnK complex (PDB- 2NS1) is presented in cartoon with monomers in yellow, green and cyan. The residues marked in red in the alignment are indicated by red sticks in the structure of one of the GlnKEc monomers**.**


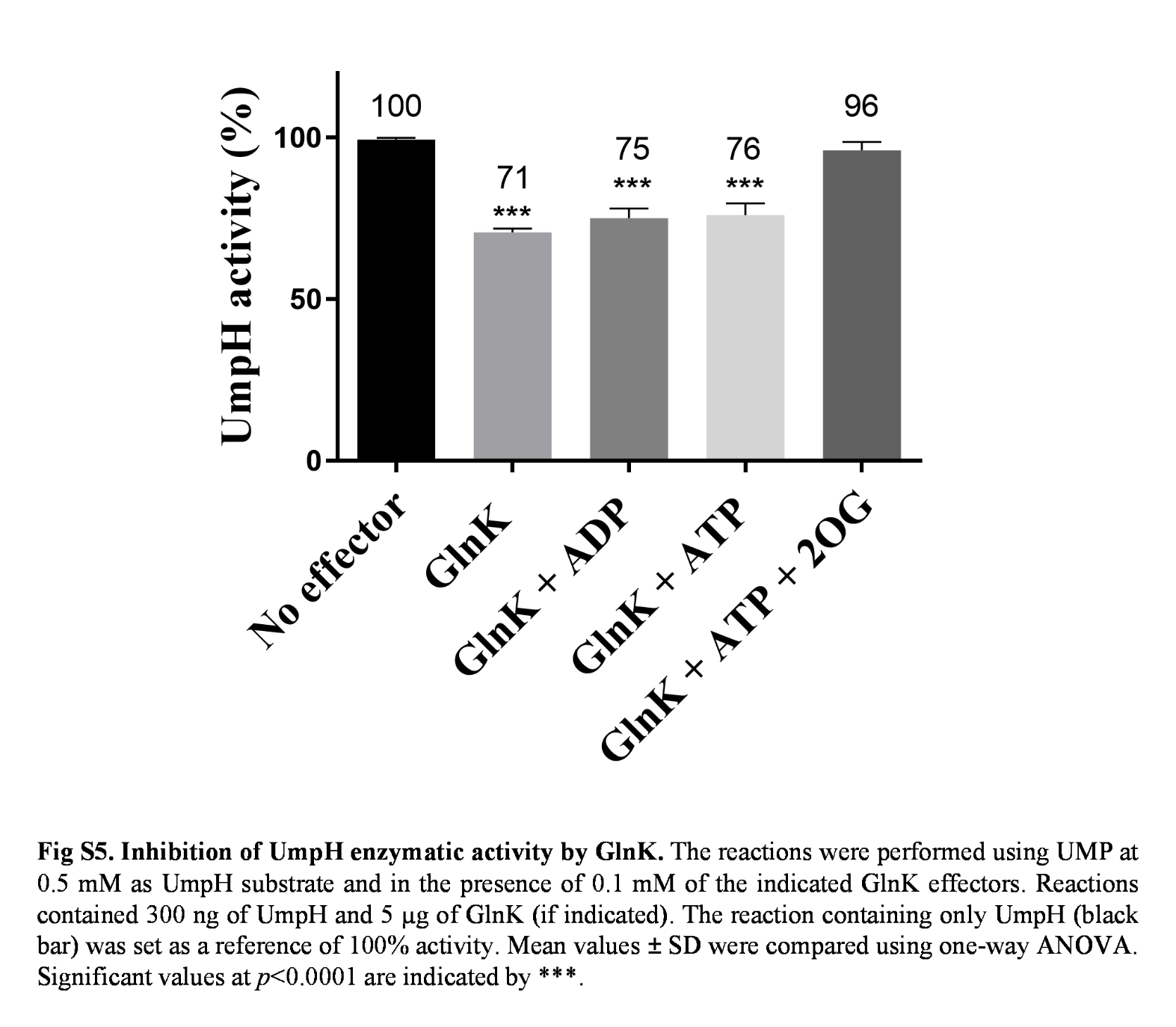


**Figure S5. Inhibition of UmpH enzymatic activity by GlnK.** The reactions were performed using UMP at 0.5 mM as UmpH substrate and in the presence of 0.1 mM of the indicated GlnK effectors. Reactions contained 300 ng of UmpH and 5 µg of GlnK (if indicated). The reaction containing only UmpH (black bar) was set as a reference of 100% activity. Mean values ± SD were compared using one-way ANOVA. Significant values at *p*<0.0001 are indicated by ***.
